# Using the Right Criteria for MCAS

**DOI:** 10.1007/s11882-024-01126-0

**Published:** 2024-01-20

**Authors:** Theo Gulen

**Affiliations:** 1 Department of Respiratory Medicine and Allergy, K85, Karolinska University Hospital Huddinge, Stockholm, SE-14186 Sweden; 2 Division of Immunology and Allergy, Department of Medicine Solna, Karolinska Institutet, Stockholm, Sweden; 3 Clinical Lung and Allergy Research Unit, Department of Medicine Huddinge, Karolinska Institutet, Stockholm, Sweden; 4 Mastocytosis Centre Karolinska, Karolinska University Hospital Huddinge, Stockholm, Sweden

**Keywords:** MCAS, Mast cell activation, Anaphylaxis, Mastocytosis, Tryptase, Hereditary alpha-tryptasemia

## Abstract

**Purpose of Review:**

The current article aims to provide a comprehensive update on diagnostic criteria for mast cell activation syndrome (MCAS), addressing challenges in diagnosing and classifying MCAS and its variants.

**Recent Findings:**

In recent years, there has been a significant increase in our knowledge regarding the underlying mechanisms responsible for the activation of mast cells (MCs) in various pathological conditions. Furthermore, a set of criteria and a classification for MCASs have been established. MCAS is characterized by the presence of typical clinical symptoms, a substantial elevation in serum tryptase levels during an attack compared to the patient’s baseline tryptase levels, and a response to MC mediator–targeting therapy.

**Summary:**

In this report, a thorough examination was conducted on the contemporary literature relating to MCAS, with a focus on comparing the specificity, sensitivity, and robustness of MCAS-related parameters within proposals for diagnosing and classifying MCAS and its variants. Moreover, the significance of employing specific consensus criteria in the assessment and categorization of MCAS in individual patients was underscored, due to the escalating occurrence of patients receiving a misdiagnosis of MCAS based on nonspecific criteria.

## Introduction

Mast cells (MCs) are granulated, multifunctional immune cells with diverse functions [1]. They can adjust their responses, depending on the stimulus encountered and the tissue in which they are stimulated [1–4]. MCs can be activated by various mechanisms, but the most common one is through the cross-linking of immunoglobulin E (IgE) molecules bound to the surface by high-affinity FcεRI receptors [5–8]. Other mechanisms that can activate MCs include the activation of surface G protein–coupled receptors such as Toll-like receptors, complement receptors C3a and C5a, and mas-related G protein receptor (MRGPRX2) [9]. When MCs are activated, they release biologically active mediators and the role of these substances in the clinical symptoms of MC disorders is heterogeneous [1–10].

The severity of symptoms related to MC activation can vary from mild to severe and even life-threatening. Furthermore, these symptoms can be either acute or chronic in nature. Acute MC activation is commonly observed in allergic reactions and can manifest as localized events specific to the affected tissue or as systemic symptoms resulting from widespread MC activation [10, 11]. Examples of tissue-specific consequences of MC activation include urticaria, allergic rhinitis, or asthma, and the symptoms, in most instances, are limited to the area of the interaction with the trigger. However, there are also instances where generalized tissue-specific symptoms occur, as seen in chronic idiopathic urticaria. Systemic activation of MCs encompasses the conditions of anaphylaxis and MC activation syndrome (MCAS) [12–14]. Furthermore, severe or even life-threatening MC activation–related events may occur when MCs are in a “hyperreactive” state and/or the burden of MC is high, as in patients with mastocytosis [15•, 16•].

The purpose of the current article is to provide a clear understanding of the main challenges encountered when diagnosing and classifying MCAS and its variations.

## Mast Cell Activation Syndrome

### Definition

Mast cell activation syndrome (MCAS) is an uncommon disorder denoted by periodic sudden-onset episodes of severe systemic symptoms, encompassing an array of disorders with multiple etiologies, whether clonal or non-clonal. These symptoms are directly associated with the excessive release of MC mediators and in most cases the episodes present as anaphylaxis [14, 15•, 16•]. MCAS is considered to be part of the spectrum of MC disorders, along with anaphylaxis and mastocytosis. However, it is important to note that while these conditions are interrelated, they are also distinct from each other.

### Diagnosis of MCAS

**Fig. 1:**
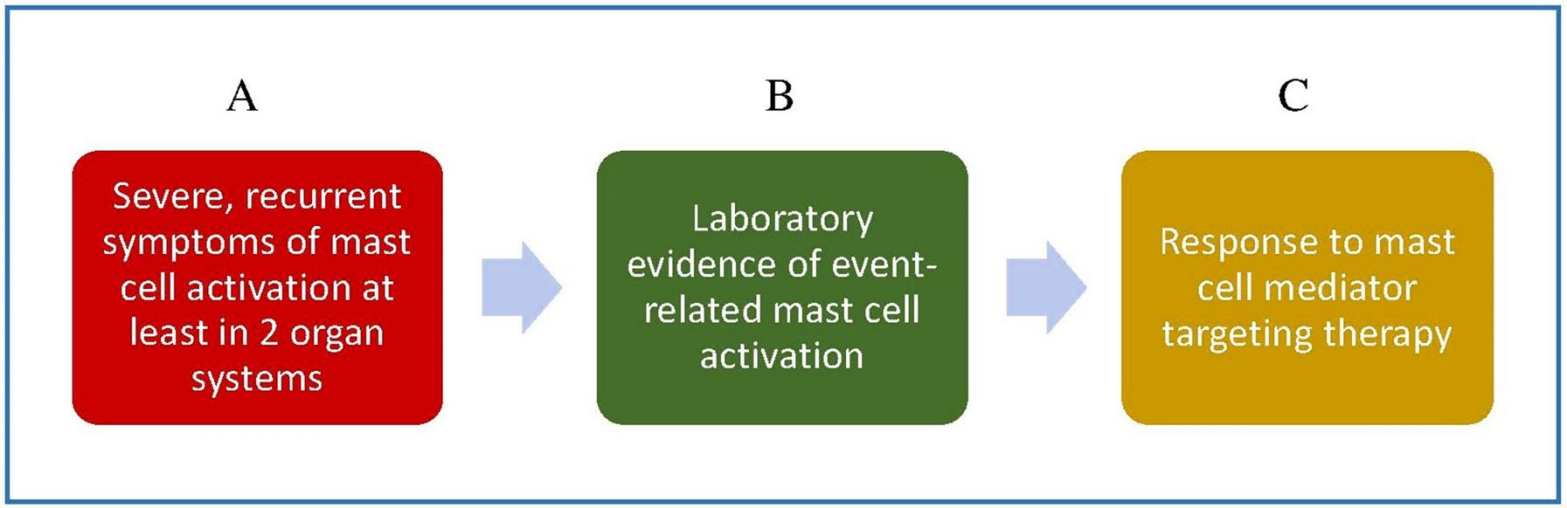
Diagnostic criteria for mast cell activation syndrome (MCAS). All three criteria must be fulfilled to confirm a diagnosis of MCAS: **A** clinical criterion, **B** laboratory criterion; **C** response criterion. Please refer to the text for further explanation

MCAS may be diagnosed when the symptoms of MC activation are systemic (involving more than one organ system), severe, and recurrent and the MCAS criteria are fulfilled [17••, 18••]. There are three sets of criteria required for an MCAS diagnosis as illustrated in Fig. 1: (1) the presence of typical, severe, episodic MC activation symptoms in ≥ 2 organ systems; (2) the detection of a substantial transient increase in a validated marker of MC activation during the symptomatic event; (3) the control of symptoms with MC mediator–targeting drugs.

The clinical criterion of MCAS requires the simultaneous involvement of ≥ 2 organ systems [17••, 18••]. Thus, MCAS events typically meet the clinical criteria of anaphylaxis. For instance, flushing and hypotensive syncope occurring simultaneously strongly suggest MCAS [19•]. When MC activation–related symptoms are severe and recurrent, the possibility of MCAS diagnosis may be considered. These symptoms encompass a range of organ systems, including the skin (urticaria, angioedema, and flushing), the gastrointestinal system (nausea, vomiting, diarrhea, and abdominal cramping), the cardiovascular system (tachycardia, hypotension, syncope), and the upper and lower respiratory systems (conjunctival injection, nasal pruritus, stuffiness, wheezing, dyspnea). Although neurological and/or musculoskeletal symptoms are commonly observed, they are not exclusive to MCAS [18••, 19•].

Secondly, the diagnosis of MCAS requires laboratory proof. Hence, the clinical symptoms of MCAS are associated with an acute, substantial increase in the levels of a validated mediator of systemic MC activation during an episode, either in serum or urine, compared with the patient’s baseline levels. Without including such biochemical markers and their event-related increase, the clinical symptomatology cannot be objectively confirmed. Currently, tryptase is the most MC-specific mediator that best fulfills the laboratory criterion and is used as a gold standard to document MC activation [20–22]. The serum tryptase level usually increases during acute events of systemic MC activation (e.g., anaphylaxis, MCAS), peaks in serum about 1 h after clinical onset of the event, and then declines with a *t*½ of about 2 h, so may remain elevated 3 h (1 *t*½), 5 h (2 × *t*½), or longer, depending on the magnitude of the initial elevation, which correlates best with the magnitude of the drop in mean arterial pressure [23–25]. Genetically determined normal serum baseline tryptase (sBT) level is generally considered < 8 ng/mL. To diagnose MCAS, the event-related tryptase should be greater than sBT * 1.2 + 2 ng/mL to confirm the clinical suspicion of MC activation, i.e., typical clinical symptoms of anaphylaxis are also present [17••, 18••, 19•]. This approach has been validated and is broadly accepted [26, 27•]. Unfortunately, there are some drawbacks in clinical practice, e.g., if acute sample collection is overlooked or delayed. If there are no previous sBT levels available, such baseline measurement should be determined in serum collected after a minimum of 24 h following the complete recovery from a suspected MC activation episode. Moreover, it should be kept in mind that a normal sBT level does not exclude MCAS, whereas a high sBT alone is not an indication or criterion of MCAS.

Mediators other than tryptase, including urinary metabolites of histamine, prostaglandin D2 (PGD2), and leukotrienes, are also available but less specific for MCs and MCAS [28, 29, 30••]. Additionally, the sensitivity and specificity of these markers have not been determined, nor have the reliable indicators of systemic MC activation, such as significant increase and cut-off levels. However, recently, it has been suggested to consider levels higher than 30% above the upper limit of normal as pathologic [18••, 30••]. Although 24-h samples of urinary metabolites are advised, shorter collection times or spot analyses are also discussed [28, 29].

Urinary metabolites of histamine have been studied and reported to correlate with MC burden and MC activation [28, 31]. N-methyl histamine and 1-methyl-4-imidazole acetic acid are the most commonly measured histamine metabolites [32–34]. Measuring plasma histamine levels as a marker of MC activation is not generally recommended, because histamine is often derived from basophils at baseline and can be influenced by a variety of factors during and after blood collection including bacterial flora of the urinary tract, storage conditions, and diet [31]. Furthermore, PGD2 is a well-known product of activated MCs [28, 35–39]. Several studies have shown that during anaphylaxis, as well as in patients with systemic mastocytosis (SM), the levels of the prostaglandin D2 metabolite 9α-11β-PGF2 in urinary samples are elevated compared to healthy controls [28, 35, 40, 41]. However, in most studies, the event-related increases of PGD2 over the individual’s baseline have not been reported. PGD2, while primarily released by MC, is also produced by other immune and nonimmune cell types [42–45]. This is important to recognize, because elevations in PGD2 might be due to a pathologic process independent of MC activation. Additionally, leukotriene C4 (LTC4) is a lipid mediator that is released during MC activation and undergoes metabolism into leukotriene D4, which is then converted to leukotriene E4 (LTE4) [46]. Urinary LTE4 was reported to be higher in patients with anaphylaxis who developed severe hypotension and also in patients with SM [41, 47–50]. Although these lipid mediator metabolites may be quite useful at ruling out MC activation when measured in urine produced during the onset and several hours after onset of the MC activation event, assays are difficult to perform and only available in a few laboratories.

Moreover, the clinical utility of serotonin, neuropeptides, heparin, platelet-activating factor (PAF), and chromogranin A (CgA) as potential biomarkers for MC activation remains unproven due to insufficient data, despite ongoing discussions [51–57]. For instance, the reported rise in plasma heparin activity following venous occlusion in patients with MC activation symptoms does not serve as sufficient validation for utilizing this test as a biomarker for MC activation [54]. Furthermore, no evidence currently exists to demonstrate a causative role of venous occlusion in MC activation. Additionally, there is currently no scientific evidence supporting CgA as a biomarker for MC activation in humans, and reported data show no elevation in CgA levels among mastocytosis patients [57].

Thirdly, the MCAS diagnosis requires a favorable response to agents that act as MC stabilizers or inhibitors of MC mediators, such as histamine receptor antagonists (H1- and H2-antihistamines), leukotriene blockers, MC stabilizers, and aspirin or non-steroidal anti-inflammatory agents (NSAIDs) [15•, 58, 59•]. A stepwise approach is recommended for treating MCAS patients. Ideally, the therapy should focus on addressing the elevated mediators and controlling symptoms with the lowest effective dose. Measuring urinary mediators during flares may help to identify the specific mediator(s) responsible for the symptoms, and therapies are expected to provide relief and decrease MC activation events. In rare cases, anti-IgE therapy or KIT-targeting kinase blockers may be required [60, 61•, 62•, 63–65].

#### Anaphylaxis Versus MCAS: Related but Not Identical

Anaphylaxis is the best recognized systemic MC activation disorder and is caused by excessive release of various MC mediators leading to a constellation of varied symptoms from different organ systems. It is an emergency condition and may potentially lead to death by airway obstruction or cardiovascular collapse, if not promptly treated. Anaphylaxis is usually considered to be a rare condition and the studies from the USA suggest an incidence of up to 40–50 people per 100,000 person-years [66–68], whereas the studies from Europe suggest a lower incidence of 1.5–7.9 per 100,000 person-years [69, 70]. Moreover, the lifetime prevalence of anaphylaxis has been estimated to be approximately 0.3% [71]. Furthermore, the food-induced anaphylaxis is the most common cause in children corresponding to 80–92% of the anaphylaxis [72], whereas Hymenoptera venom– or drug-induced anaphylaxis is dominating elicitors among adults [73].

**Fig. 2:**
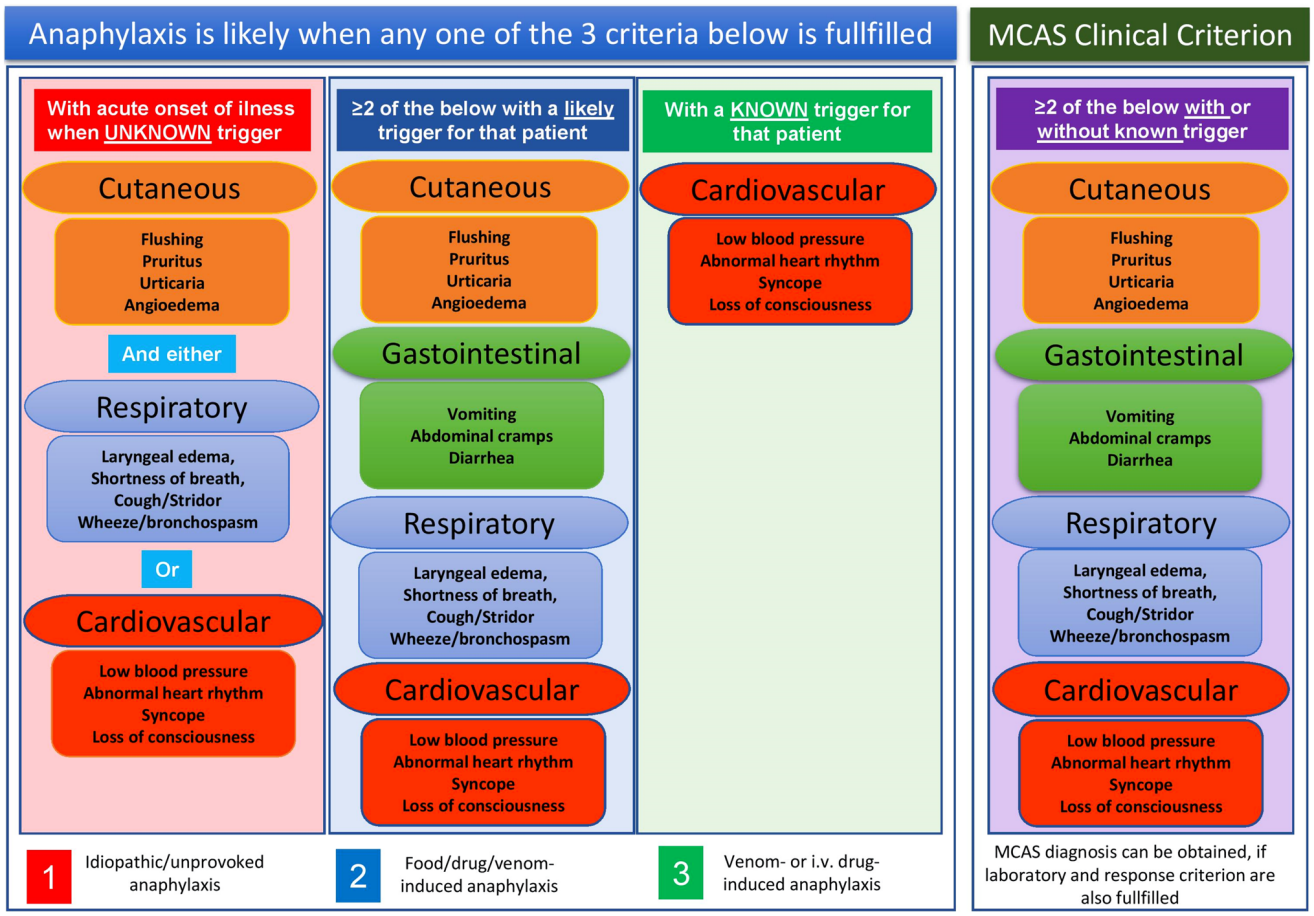
Comparison of diagnostic criteria of anaphylaxis and clinical criterion for MCAS [18••, 74]. Please see the text for detailed discussions

The diagnosis of anaphylaxis may be challenging and the line differentiating an allergic reaction from anaphylaxis is not always easily discernible. According to international consensus on the clinical criteria, the diagnosis of anaphylaxis requires concurrent occurrence of symptoms from minimum two organ systems that are related to the cutaneous, gastrointestinal, respiratory, and cardiovascular systems [74]. These criteria have been widely adopted and both retrospectively [75] and prospectively [76] validated. The required organ system involvement varies depending upon whether there is a “likely” or “known” trigger for the actual patient. Exceptionally, in context of confirmed allergy (e.g., insect venom, drug) for the given patient, an anaphylaxis diagnosis can be made only by cardiovascular system involvement (hypotension and/or syncope) after re-exposure. Additionally, even when there is no likely cause of the reactions, as in unprovoked anaphylaxis, when the onset of illness is acute, a diagnosis of anaphylaxis can be made when either reduced blood pressure (or associated symptoms, such as syncope) and/or respiratory compromise or laryngeal edema is present accompanied by the involvement of the skin–mucosal tissue symptoms (Fig. 2) [74].

Thus, anaphylaxis and MCAS are interrelated, but two distinct conditions. Patients with anaphylaxis are the archetype of MCAS; however, not all anaphylaxis episodes fulfill the diagnostic criteria of MCAS. In order to be qualified as MCAS, it also requires that these clinical reactions are recurrent (at least two episodes) as well as laboratory and response criterion are also fulfilled. Thus, not all anaphylaxis episodes can be classified as MCAS. Likewise, not all MCAS episodes reach the severity of anaphylaxis and fulfill the criteria of anaphylaxis. For instance, in patients with unprovoked episodes of MC activation, concomitant appearance of cutaneous and GI symptoms can be considered the clinical criterion of MCAS; nevertheless, to classify this as unprovoked/idiopathic anaphylaxis, in addition to above-mentioned symptoms, either respiratory or cardiovascular system involvement is required. Hence, not all MCAS episodes fulfill the criteria of anaphylaxis [77•]. Figure 2 illustrates the clinical criteria of anaphylaxis in different context and compares those to clinical criterion of MCAS.

Hence, all above-mentioned three criteria should be fulfilled to confirm a diagnosis of MCAS. Moreover, the documented presence of clonal MCs alone or the diagnosis of cutaneous mastocytosis (CM) or SM alone is not an indication of MC activation, even though such patients are susceptible to severe MC activation–related events that could lead to the diagnosis of MCAS. Additionally, increased levels of sBT or other MC mediators alone should not be employed to diagnose MCAS, if an “event-related” transient increase (from the patient’s baseline) of a specific MC mediator is not confirmed. For instance, tryptase levels may be elevated in unrelated conditions, such as hypereosinophilic syndromes, myelodysplastic syndrome, helminth infestation, end-stage kidney disease, and hereditary alpha-tryptasemia (HαT). Furthermore, given that the clinical symptoms often ascribed to MCAS are not consistently distinct for MC activation, it becomes crucial to establish their association with MC activation. Otherwise, most patients with SM and many with unrelated disorders would be misdiagnosed with MCAS.

### Classification of MCAS

Upon confirmation of an MCAS diagnosis, further classification becomes imperative resulting in the categorization of MCAS into three principal variants with varying mechanisms that activate MC in different MCAS phenotypes [17••, 18••].

#### Primary “Clonal” MCAS

In some instances, patients suffering from MCAS may display coexisting clonal MCs within the bone marrow, as seen in both mastocytosis (SM or CM) or monoclonal mast cell activation syndrome (MMAS) [17••, 18••]. Additionally, patients presenting with clonal MC disorders commonly exhibit varying extents of expansion in the MC compartment derived from a progenitor carrying a genetic defect that presumably reduces the cell’s threshold for activation [15•, 16•]. These patients may have elevated sBT levels, carry *KIT* D816V mutations in lesional MCs, or have other markers of MC clonality, such as aberrant CD25 expression. Such MCAS patients are considered to have primary (i.e., clonal) MCAS and its diagnosis can only be made after an extracutaneous biopsy, most often after a bone marrow biopsy [17••, 18••]. Thus, patients with clonal MCAS are required to fulfill the diagnostic criteria of both MCAS and clonal MC disease as below.

##### Mastocytosis

Mastocytosis encompasses a complex heterogeneous multisystem disorder characterized by a pathologic activation and accumulation of clonally aberrant MCs in one or more organs, including the skin, bone marrow, and gastrointestinal tract [78, 79•, 80••]. It is a rare condition, and its prevalence is estimated to be 1 in 10,000 persons in recent studies [81–84]. In general, mastocytosis can be divided into two main categories: CM and SM involving at least one additional organ than the skin. CM is the main form of the disease in children and the most common form of skin involvement is maculopapular cutaneous lesions, also known as urticaria pigmentosa (UP). The majority of children have a benign course and experience spontaneous improvement; however, patients with adult-onset mastocytosis have a persistent disease and may or may not present with skin lesions. According to the WHO diagnostic criteria, the diagnosis of SM requires the existence of a major and a minor criterion or three minor criteria on extracutaneous biopsy materials, most commonly from the bone marrow (see Table 1) [78, 79•, 80••]. In the majority of adult patients with UP, which is known as mastocytosis in the skin (MIS), MC infiltrates are also found in the bone marrow, corresponding to the final diagnosis of SM [79•]. Moreover, SM has been classified into several subgroups, with more than 85% of affected subjects having indolent SM (ISM) with a good prognosis [83, 84]. The remaining 15% of affected subjects have more aggressive variants, i.e., advanced SM (including aggressive SM, SM with associated hematologic neoplasm, and MC leukemia) with a poor prognosis [78, 79•].

The clinical course of SM varies greatly, ranging from asymptomatic disease to a highly aggressive course. Patients with ISM may exhibit various clinical symptoms such as flushing, itching, rapid heartbeat, dizziness, low blood pressure, fainting, breathing difficulties, abdominal pain, nausea, vomiting, diarrhea, headache, lethargy, fatigue, impaired concentration, irritability, anxiety, depression, arthralgia, myalgia, and osteoporosis due to the local or remote effects of MC mediators [78]. However, not every patient exhibits every one of these symptoms, so the reason for this heterogeneity remains unclear. Nevertheless, a history of flushing is a cardinal symptom. Moreover, some subjects may experience isolated symptoms, whereas others develop a constellation of signs and symptoms indistinguishable from those of anaphylaxis [85, 86]. Typically, patients suddenly feel very warm and then experience palpitations, dizziness, and a decrease in blood pressure due to systemic vasodilatation that often leads to syncope [50]. Acute attacks may be brief or prolonged, but duration is usually 15 to 30 min [50]. Patients often experience severe fatigue lasting around 24 h following the spells [50]. Triggers vary greatly among patients and include physical exertion, cold, heat, insect venoms, consumption of alcohol, infections, non-steroidal anti-inflammatory drugs (NSAIDs), and emotional stress. The mediator levels do not usually show clear association with the clinical phenotypes, although the baseline levels of mediators including tryptase, histamine, and PGD2 are generally elevated [28].

Notably, anaphylaxis is less common in subjects with advanced SM compared to patients with ISM. These patients commonly experience symptoms related to MC infiltration and uncontrolled accumulation, such as cytopenia, hepatosplenomegaly, lymph adenopathy, osteolytic bone lesions, and liver dysfunction [78].

**Table 1:** Diagnostic criteria of SM and MMAS [78, 79•]

**SM**	**Diagnosis is confirmed if the patient exhibits one major criterion and one minor criterion or exhibits three of the four minor criteria in extracutaneous organ biopsy specimens**
**Major criterion**	1. Multifocal aggregates of MCs (≥ 15 MCs per cluster) in biopsy sections
**Minor criteria**	1. In MC infiltrates in extracutaneous biopsy sections, > 25% of the MCs (CD117+) are spindle-shaped or have atypical morphology 2. Presence of an activating KIT mutation at codon 816, generally D816V, in bone marrow, blood, or other extracutaneous organ(s) 3. Detection of aberrant MC clones expressing CD117 with CD25 and/or CD2 and/or CD30 in bone marrow or blood or another extracutaneous organ(s) 4. Baseline serum tryptase persistently exceeds ≥ 20 ng/mL
**MMAS**	**Diagnosis requires presence of one or two of the following minor criteria of SM**
**Minor criteria**	1. Presence of an activating KIT mutation D816V, in bone marrow, blood, or other extracutaneous organ(s) AND/OR 2. Detection of aberrant MC clones expressing CD117 with CD25 in bone marrow or blood or another extracutaneous organ(s)

*SM* Systemic mastocytosis, *MMAS* monoclonal mast cell activation syndrome

##### Monoclonal Mast Cell Activation Syndrome

Monoclonal mast cell activation syndrome (MMAS) is a newly recognized variant of clonal MC disorders and is characterized by severe episodes of anaphylaxis with hypotensive syncope in affected patients [87, 88]. While patients diagnosed with MMAS present detectable clonal MCs expressing the D816V mutation and/or CD25 + aberrant markers, they do not meet the diagnostic criteria established by the WHO for classifying as having SM (Table 1). Moreover, the sBT levels of these patients generally show a slight elevation, often ranging between 10 and 20 ng/mL. Additionally, they lack typical skin changes of mastocytosis (MIS).

#### Secondary MCAS

The majority of patients with symptoms caused by intermittent, recurrent MC activation typically have non-clonal etiologies. These patients, known as secondary MCAS, experience symptoms associated with MC activation due to the involvement of both IgE-mediated (such as food-, drug-, or Hymenoptera venom–induced anaphylaxis) and non-IgE-mediated (e.g., exercise) mechanisms.

#### Idiopathic MCAS

In certain cases, a patient who undergoes severe, recurrent MC activation may have an unremarkable work-up for allergic causes and exhibit no indication of clonal MC disease (typically ruled out following a bone marrow examination). These patients are evaluated for either IA or idiopathic (non-clonal) MCAS diagnoses, depending on the criteria they meet [77•].

Furthermore, over recent years, an increasing number of studies have indicated that multiple variants of MCAS can coexist within the same patient, putting these patients at the greatest risk for the development of life-threatening MCAS episodes [15•, 16•]. For instance, in patients with SM and an IgE-dependent allergy against bee or wasp venom, both the underlying SM and the underlying venom allergy may act as a trigger of anaphylaxis and thereby induce a mixed (primary + secondary) form of MCAS. Hence, such *combined* (mixed) form of MCAS should always be considered in patients with severe anaphylaxis. In general, the signs and symptoms of recurrent IgE-mediated anaphylaxis may be the initial presentation of secondary or combined MCAS, whereas IA can consist of the initial symptoms of clonal or idiopathic MCAS.

### Hereditary Alpha-Tryptasemia

More recently, additional genetic features have been linked to an elevated risk to develop anaphylaxis and MCAS. One such factor is HαT, a genetic trait defined by extra copies of the TPSAB1 gene encoding for alpha-tryptase, and an elevated sBT concentration in most carriers [89–91]. Patients with HαT are generally measured to have sBT levels higher than 8 ng/mL (often > 10 ng/mL) [90]. HαT is found in approximately 6% of the general population and the majority of individuals with HαT appear to be asymptomatic [92, 93•].

However, HαT has been linked to an increased prevalence of SM and an increased risk of severe mediator-related symptoms and MCAS in those with SM [91, 94•, 95•]. Furthermore, HαT is more prevalent in those with ISM than advanced SM. Moreover, a coexisting IgE-dependent allergy, such as an insect venom allergy, is frequently observed in carriers of HαT with SM and MCAS [91, 94•, 95•]. Hence, these patients apparently are the highest risk to develop severe or even life-threatening anaphylaxis or even a combined form of MCAS [91, 94•, 95•]. Presently, however, whether a pure form of hereditary (HαT +) MCAS indeed exists is under debate. Therefore, HαT is currently thought to be a modifying factor that may influence the prevalence and severity of anaphylaxis.

### When a Bone Marrow Investigation Should Be Performed

**Fig. 3:**
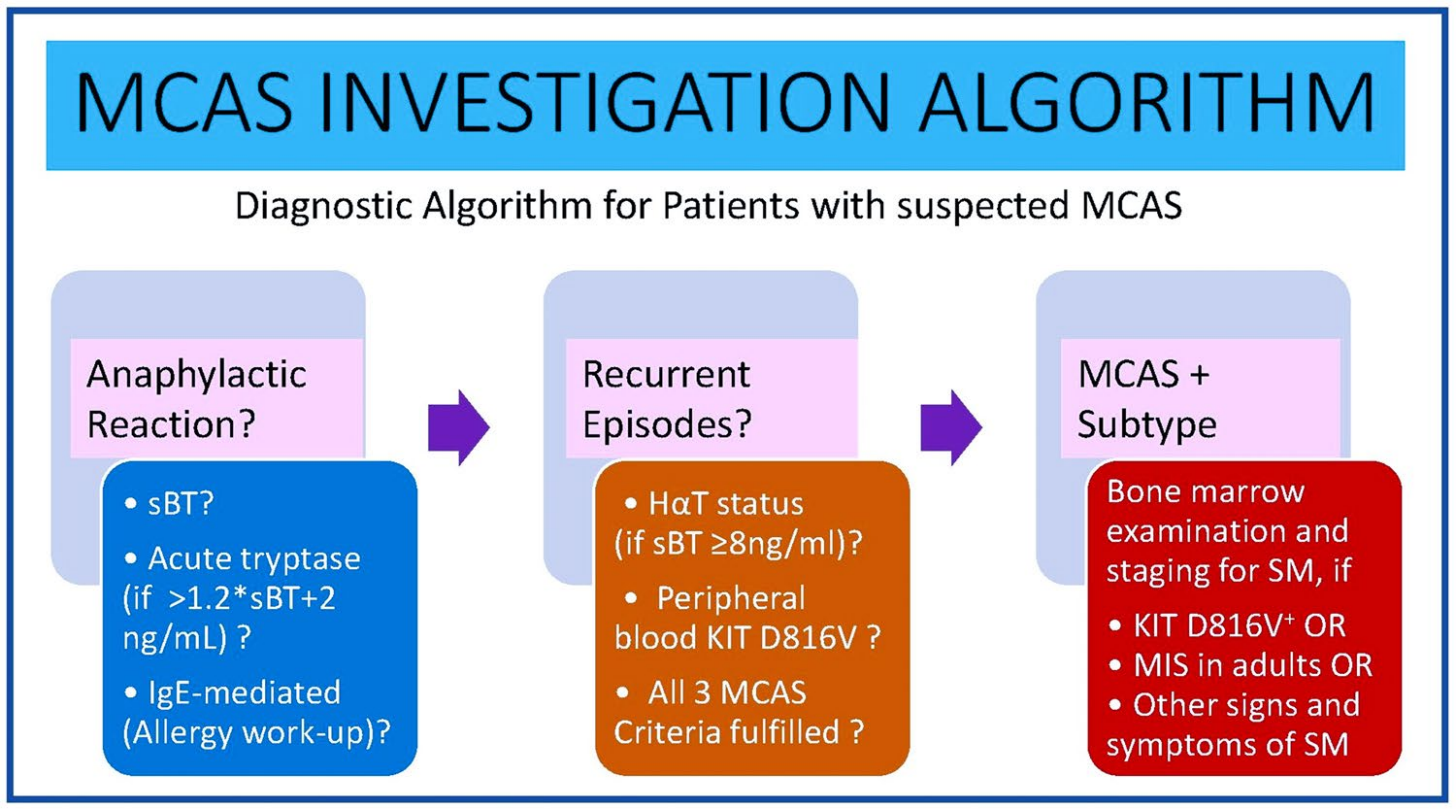
Diagnostic algorithm for patients with suspected MCAS. Investigations should be adjusted to the case history of individual patients. To establish the diagnosis of MCAS, all three MCAS criteria must be fulfilled. Abbreviations: sBT, serum baseline tryptase; HαT, hereditary alpha-tryptasemia; SM, systemic mastocytosis; MIS, mastocytosis in the skin

A BM examination should be considered after an initial screening which includes a thorough physical examination with skin inspection, blood counts, serum chemistry, and a sBT level, as well as peripheral blood testing for *KIT* D816V (Fig. 3). Moreover, if available, tryptase genotyping is recommended for the patients with sBT ≥ 8 ng/mL. If *KIT* D816V is detectable in an adult patient, a BM examination should be conducted regardless of the sBT and HαT status [18••]. Nevertheless, if KIT D816V mutation is not detected but HαT is found, BM investigations are not necessary unless there are other features that could suggest the presence of SM. Furthermore, in cases where a symptomatic patient presents recurring episodes of anaphylaxis and all these variables demonstrate negative findings, the application of predictive tools such as the Spanish Network on Mastocytosis (Red Española de Mastocitosis [REMA]) score [96], Karolinska score [97], or National Institutes of Health Idiopathic Clonal Anaphylaxis Score [98] becomes essential to estimate the probability of the patient having clonal MC disorder. This is particularly important in symptomatic patients who lack typical skin lesions of mastocytosis.

## MCAS Mimickers

**Fig. 4:**
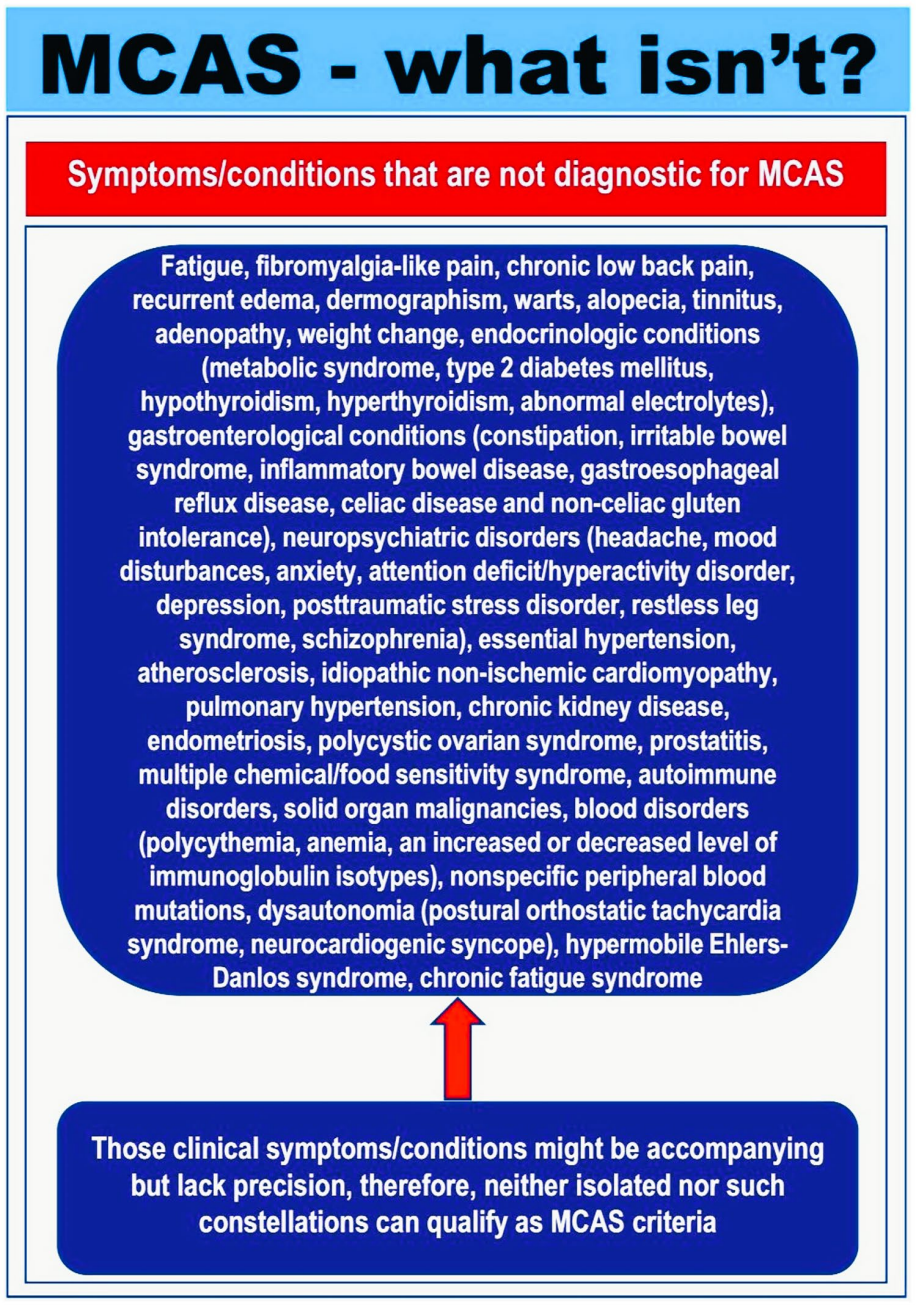
Symptoms and conditions mimicking mast cell activation and MCAS [18••]. When patients do not present with the typical symptoms of anaphylaxis or with chronic rather than episodic acute symptoms, it may be particularly challenging for the clinicians to establish a diagnosis. This is because most symptoms attributable to MC activation are not MC-specific but can also be found in other clinical conditions and disorders. Moreover, patients with multiple chemical, environmental, or food intolerances should not be diagnosed with MCAS if they do not meet the criteria

Even though an international consensus group has set diagnostic criteria and classification for MCAS in the past decade [17••, 18••, 19•], debates regarding the usage of the term MCAS in different patient categories persist, leaving unresolved controversies. A significant difficulty lies in the fact that a considerable number of patients, whose symptoms are believed to be caused by MC activation, incorrectly receive a diagnosis of MCAS without any substantial proof that their clinical manifestations and symptoms are indeed derived from MC activation and mediator release [17••, 18••, 19•]. Among these patients, there could be those who may suffer from MC activation disorders (MCADs) or non-specified MC activation reactions [80••]. In these patients, localized MC activation, milder MC activation, or MC activation possibly involving a limited set of mediators or only one organ system may be implicated. Furthermore, some of the attributed symptoms may not even be related to MC activation [80••]. This is because most symptoms attributable to MC activation, including isolated flushing, pruritus, headache, abdominal pain, or tachycardia, are not MC-specific but can also be found in other clinical conditions and disorders (Fig. 4) [99–105].

Additionally, a number of other conditions, such as cardiovascular pathologies (myocardial infarction, myocarditis), endocrinologic diseases (e.g., adrenal crisis, thyroid disease, estrogen or testosterone deficiency, adrenal insufficiency, carcinoid), vocal cord dysfunction, cutaneous diseases (e.g., atopic or contact dermatitis, rosacea), neurologic disorders (e.g., seizures, stroke, multiple sclerosis, meningitis, dysautonomia, vasovagal syncope), psychologic disorders (e.g., panic attacks and anxiety, depression), gastrointestinal diseases such as an inflammatory bowel disease, and infections (septicemia), can mimic symptoms of MCA. It is important to note that some of these patients may easily be misdiagnosed as having MCAS when applying less stringent criteria, but not the criteria adopted by the Vienna consensus group [17••, 18••]. Hence, a broad differential diagnosis should be considered before a diagnosis of MCAS is made when evaluating patients with suspected MC activation [17••, 18••, 19•, 106]. This process may take longer than predicted, particularly in patients with idiopathic MCAS (iMCAS), and the diagnostic delay may be approximately 4 years (according to personal experience).

Furthermore, it should also be mentioned here that studies indicating the true prevalence of MCAS using evidence-based diagnostic criteria have been lacking until recently. A newly published study investigated the prevalence and clinical characteristics of patients with iMCAS among 703 adult consecutive patients referred due to suspected mast cell disorders [107]. Interestingly, of the investigated patients, 35% patients had at least one episode of anaphylaxis. An overall prevalence of iMCAS in this study was 4.4%, with a relatively higher prevalence among patients with unprovoked anaphylaxis (27%) [107]. This study supports the notion that anaphylaxis is the archetype of MCAS and that MCAS is an uncommon condition. Thus, clinicians should be cautious when diagnosing iMCAS to avoid misdiagnosis.

## Concluding Remarks

Typically presenting as anaphylaxis, MCAS encompasses a severe systemic reaction caused by the acute release of MC mediators. When patients do not present with the typical symptoms of anaphylaxis, MCAS is much less likely to be the correct diagnosis. Because, in the majority of these patients, there is only a slight increase or no increase at all in serum tryptase levels compared to the individual’s baseline [17••, 18••, 19•]. Even the other validated markers of MC activation may not even show increases in biological fluids in these patients. Consequently, these cases often pose a clinical challenge, and it may be hard to rule out the involvement of other inflammatory effector cells such as basophils or eosinophils. Hence, the ultimate diagnosis remains descriptive, as MC involvement can only be speculated.

Understanding the category of MCAS and its underlying etiology should provide a solid basis for establishing a personalized treatment plan for MCAS patients. A stepwise, individual-based approach in pharmacotherapy options appears to be the most convenient strategy [58, 59•]. Acute episodes of any variant of MCAS should be promptly treated with intramuscular epinephrine [108–111]. Allergen immunotherapy is recommended for MCAS patients with documented Hymenoptera venom allergy, and in patients with mixed MCAS, life-long immunotherapy is standard [112••].

Finally, additional research is required to identify novel biomarkers of MC activation, enabling healthcare providers in differentiating between genuine cases of MCAS and its mimickers, especially in patients with milder or chronic or localized symptoms who do not meet the Vienna consensus criteria for MCAS [17••, 18••].

## Data Availability

No datasets were generated or analysed during the current study.
